# Multimodal assessment of communicative-pragmatic features in schizophrenia: a machine learning approach

**DOI:** 10.1038/s41537-021-00153-4

**Published:** 2021-05-24

**Authors:** Alberto Parola, Ilaria Gabbatore, Laura Berardinelli, Rogerio Salvini, Francesca M. Bosco

**Affiliations:** 1 Department of Linguistics, Semiotics and Cognitive Science, Aarhus University, Aarhus, Denmark; 2 Department of Psychology, University of Turin, Turin, Italy; 3 Department of Mental Health, A.S.L. “Città di Torino”, Turin, Italy; 4 Instituto de Informática, Universidade Federal de Goiás, Goiânia, GO Brasil; 5 Centro Interdipartimentale di Studi Avanzati di Neuroscienze–NIT, University of Turin, Turin, Italy

**Keywords:** Schizophrenia, Biomarkers, Human behaviour

## Abstract

An impairment in pragmatic communication is a core feature of schizophrenia, often associated with difficulties in social interactions. The pragmatic deficits regard various pragmatic phenomena, e.g., direct and indirect communicative acts, deceit, irony, and include not only the use of language but also other expressive means such as non-verbal/extralinguistic modalities, e.g., gestures and body movements, and paralinguistic cues, e.g., prosody and tone of voice. The present paper focuses on the identification of those pragmatic features, i.e., communicative phenomena and expressive modalities, that more reliably discriminate between individuals with schizophrenia and healthy controls. We performed a multimodal assessment of communicative-pragmatic ability, and applied a machine learning approach, specifically a Decision Tree model, with the aim of identifying the pragmatic features that best separate the data into the two groups, i.e., individuals with schizophrenia and healthy controls, and represent their configuration. The results indicated good overall performance of the Decision Tree model, with mean Accuracy of 82%, Sensitivity of 76%, and Precision of 91%. Linguistic irony emerged as the most relevant pragmatic phenomenon in distinguishing between the two groups, followed by violation of the Gricean maxims, and then extralinguistic deceitful and sincere communicative acts. The results are discussed in light of the pragmatic theoretical literature, and their clinical relevance in terms of content and design of both assessment and rehabilitative training.

## Introduction

Pragmatics is classically defined as the ability to use language to convey a specific communicative meaning in a given context^1–3^. More recently, this definition has also included the use of other expressive means such as non-verbal/extralinguistic modalities, e.g., gestures and body movements, and paralinguistic cues, e.g., prosody and tone of voice^4^. Schizophrenia is associated with a specific language impairment^5–7^. Patients’ difficulties refer to different levels of language processing, ranging from phonological aspects to word and semantic production^8–10^. The term schizophasia was coined to include some of these difficulties, including clanging, neologism, and unintelligible utterances^11–13^. However, several studies have also pointed out that communicative difficulties may persist even when the syntactic and semantic abilities of patients with schizophrenia are preserved^14–16^.

In the current literature, a well-established notion is that patients with schizophrenia show pervasive difficulties in terms of communicative-pragmatic ability^17–22^. Pragmatic ability relies on inferential processes in order to fill the gap that often exists between the literal meaning of an utterance and what the speaker actually intends to communicate as, for example, in the statement “What a nice person!” referring to an individual acting very impolitely. A large number of studies have pointed out that patients with schizophrenia specifically have more difficulty than healthy controls with the comprehension of non-literal language^23^. Non-literal language refers to those communicative acts that imply a gap between the literal and the intended meaning: this is the case of indirect speech acts^24^, as well as figurative expressions like irony^25–28^, metaphors, idioms, and proverbs^29–33^. Furthermore, patients with schizophrenia may also encounter communicative difficulties when having to deal with other pragmatic phenomena, such as recognizing and repairing communicative failures^34^, as well as understanding deceit^35^. Moreover, patients with schizophrenia may often display difficulties in detecting violations of the Gricean maxims of communication^28,36^. Gricean maxims, i.e., quantity (violated by providing extra and redundant details), quality (violated when a speaker says something that is obviously false), relation (violated when the information that is provided is not related to the context of the communicative interaction) and manner (violated when the speaker uses expressions that are rude and inappropriate), represent those communicative rules to which communicative partners adhere, in order to make their communicative contributions effective and ensure a meaningful exchange of information^37^. Other difficulties have been reported in tasks assessing narrative ability^38^ and conversational skills^39,40^.

Non-verbal/extralinguistic expressive behavior, though less investigated than linguistic ability, is also impaired in schizophrenia, and represents a characterizing element of the disease^41^. Such difficulties range from perception, comprehension, and production of communicative gestures^42–44^ to facial expression recognition (for a review see refs. ^45–47^).

Moreover, patients with schizophrenia display atypical prosodic patterns, in terms of flat intonation, increased pauses, distinctive tone, and abnormal voice quality^48,49^. Previous studies indicated slower speech^50^, more pronounced pauses^51,52^, and reduced prosodic variability^53,54^. A recent meta-analysis^55^ confirmed that voice atypicalities, especially those related to duration and pitch variability measures, represent a characteristic feature of schizophrenia. Further, the ability to recognize linguistic and emotional prosodic cues has also been reported to be impaired in schizophrenia^56–58^, as shown, for example, by a difficulty in decoding sarcasm based upon voice tone^59^.

Despite this evidence, few previous studies have attempted to provide a multimodal assessment of communicative-pragmatic ability in schizophrenia, evaluating different communicative phenomena expressed through different expressive modalities within the same experimental sample. Among these, Meilijson and colleagues^60^ tested verbal, non-verbal, and paralinguistic aspects of conversation to evaluate communicative performance across different clinical populations. The authors pointed out that participants with schizophrenia performed less well than controls in all the expressive modalities. Linscott^40^ investigated communicative-pragmatic performance in patients with schizophrenia by assessing linguistic conversational ability and non-verbal aspects, i.e., those expressive behaviors used to facilitate the listener’s engagement. The authors reported that patients with schizophrenia demonstrated a higher level of pragmatic impairment as compared to healthy controls. More recently, Pawelczyk and colleagues^61^ tested different aspects of pragmatic ability in individuals with schizophrenia, i.e., inferential meaning (implicit understanding), lexical-semantic processing, written metaphors, picture-metaphors, humor, discourse analysis, emotional and linguistic prosody. The results showed that, as compared to controls, patients experienced difficulties in almost all the tasks investigated, namely, in the comprehension of implicit information and humor, in processing lexical-semantic information, in emotional and linguistic prosody, and in the discourse test. However, individuals with schizophrenia and controls performed equally well in understanding written and depicted metaphors. This last finding, though, is in contrast with the results of Deamer and colleagues^62^, who found that, in a picture metaphor comprehension task, when the experimental material is presented as a picture, and the answer does not require the use of language (i.e., choosing the correct answer from among different images), patients with schizophrenia perform less well than healthy controls. Colle and colleagues^20^ assessed the ability of individuals with schizophrenia to comprehend and produce different types of pragmatic phenomena, such as sincere, direct, and indirect communicative acts, deceit and irony, and, at the same time, investigated the linguistic and extralinguistic expressive modalities. The results of the study indicated that participants with schizophrenia performed significantly worse than healthy controls in all the tasks investigated, involving the use of both the linguistic and extralinguistic modalities. Considered as a whole, the above-mentioned studies provide a clear picture of communicative-pragmatic deficits in patients with schizophrenia; further, this evidence clearly suggests that such difficulties concern not only the linguistic modality, but other expressive modalities as well, such as the non-verbal/extralinguistic and paralinguistic modalities.

Meta-analytic evidence has demonstrated the presence of large differences between patients with schizophrenia and healthy controls in the various communicative-pragmatic domains. Parola and colleagues^63^ found a large difference between patients and controls in the comprehension of indirect speech acts (Cohen’s *d* = −0.70, *p* = 0.09), deceit (*d* = −0.92, *p* < 0.001), irony (*d* = −1.22, *p* < 0.001) and violations of Gricean maxims (*d* = −1.33, *p* < 0.001). As far as the recognition of emotional and linguistic prosodic cues is concerned, different meta-analyses have shown large differences for emotional prosody (Hoekert et al.^56^: *d* =−1.24, CI = − 1.55 to −0.93; Lin et al.^64^: *d* = −0.95, CI = −0.80 to −1.11). As for vocal expression, previous meta-analytic evidence^21,56^ has pointed out large differences in the qualitative rating of emotional prosody (*d* = −1.11, CI = − 1.78 to −0.43), and less robust differences for quantitative acoustic measures (*g* = −0.55 for pitch variability, *g* between −0.75 and −1.89 for proportion of spoken time, speech rate, and pauses). However, most of the previous studies used pragmatic tasks for assessing linguistic and prosodic ability, while the gestural modality received less attention and thus no meta-analytic evidence exists for gesture recognition and gesture production. As a whole, this evidence confirms that the impairment in the different communicative-pragmatic domains in schizophrenia is severe and widespread, with large differences between patients and controls in the various pragmatic domains.

Although previous literature clearly indicated that communicative disorder is a core deficit in schizophrenia, it remains unclear which communicative features, i.e., which pragmatic phenomena expressed via different expressive modalities, are the most informative for discriminating between patients with schizophrenia and healthy controls. In part, this reflects the complexity of communicative-pragmatic ability. Indeed, pragmatic deficits can vary as a function of the different tasks used to assess the ability and the specific pragmatic phenomena evaluated. Moreover, deficits can affect different expressive modalities, and it is thus important to provide a multimodal assessment of communicative-pragmatic ability. However, no previous studies have provided a comprehensive assessment of communicative-pragmatic ability with the primary aim of identifying the specific communicative phenomena and expressive modalities able to best discriminate between patients with schizophrenia and healthy controls. The identification of the communicative-pragmatic phenomena and the communicative expressive modalities in which patients experience greater difficulty could be a valuable aid for clinicians throughout the diagnostic procedure, helping them to identify the disease during the early stages of evaluation and improving the effectiveness of rehabilitative treatment specifically focused on these features^65,66^.

The aim of the present research is to identify the pragmatic features—communicative phenomena expressed through different communicative modalities, i.e., linguistic, extralinguistic, and paralinguistic—that are the most informative for discriminating between patients with schizophrenia and healthy controls. For this purpose, we assessed the abilities of patients with schizophrenia and healthy controls in a wide range of communicative-pragmatic phenomena, i.e., basic speech acts (statements, questions, commands, and orders^67^), sincere (direct and indirect), ironic and deceitful communicative acts, violation of the Gricean maxims, prosodic mismatch, social appropriateness, conversational ability (turn-taking and adherence to the topic). Compared to our previous studies^18,21^, this research analyzes a wider range of pragmatic phenomena, as well as additional expressive means, i.e., paralinguistic modalities. Furthermore, the present study focuses on a new research question, i.e., the identification of the communicative phenomena and expressive modalities that more reliably discriminate between patients with schizophrenia and healthy controls, and to this aim it applies a different methodology, i.e., a Machine Learning (ML) approach, and specifically Decision Tree (DT) analysis. DT analysis can be used to find the pragmatic features that best separate the data into the two groups, i.e., individuals with schizophrenia and healthy controls. This ML technique has several advantages compared to regression models as, unlike linear regression methods, it can handle nonlinear interactions between multiple predictors. Further, it provides an intuitive and intelligible representation (tree diagram) of which variables combined with which configuration can better predict the outcome, i.e., belonging to the schizophrenia vs. healthy controls groups. In this way, we aim to move a step further also with respect to our previous work^21^, by providing a more detailed picture of the communicative profile of patients with schizophrenia, and exploring which communicative phenomena, expressed using multiple communication modalities, best discriminate between patients with schizophrenia and healthy controls.

On the basis of the previous meta-analytic evidence, we hypothesize that the most informative communicative-pragmatic phenomena for distinguishing between patients with schizophrenia and controls are irony, Gricean maxims, and recognition of prosodic cues. However, no meta-analytic evidence is available for the comprehension and production of communicative acts expressed using the gestural modality, and no predictions can be made for these phenomena. Further, no previous studies have directly compared a wide range of communicative-pragmatic phenomena expressed using different communicative modalities. Thus, this study also has an explorative aim and intends to provide an initial basis for the identification of the multimodal communicative-pragmatic features which best discriminate between patients with schizophrenia and healthy controls.

## Results

### Assessment of pragmatic abilities in schizophrenia using a DT model

**Fig. 1:**
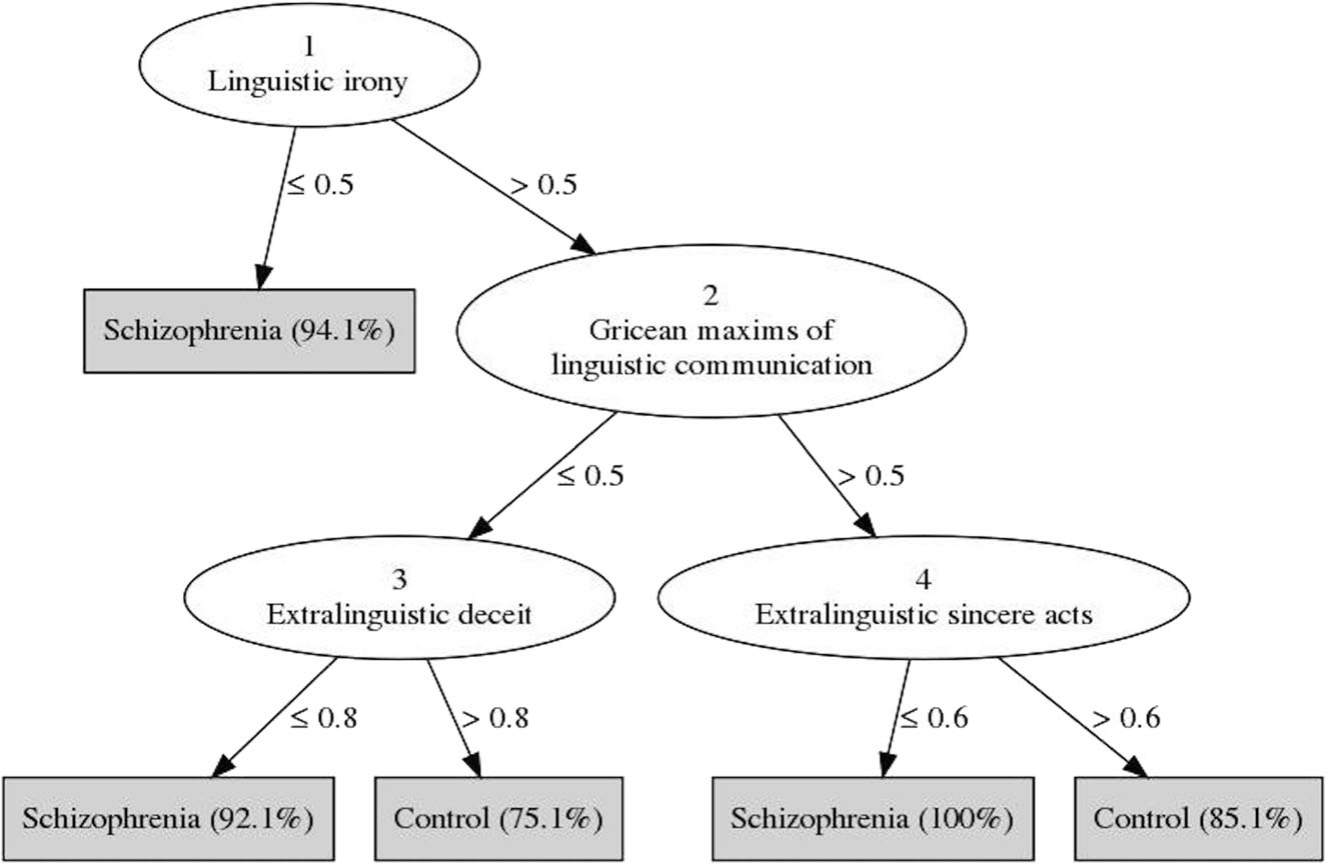
DT model. A decision tree is used to classify an example by starting at the root of the tree (testing the value of Linguistic irony) and moving through it (testing the other features) until a leaf node (gray squares), which provides the classification of the instance (schizophrenia or healthy controls).

Figure 1 shows the DT model generated. The leaf nodes (gray squares) represent the classes (schizophrenia or healthy controls), and the number in parentheses indicates the expected likelihood of new cases being classified as patients with schizophrenia or healthy controls after going through the previous decision nodes, i.e., after performing a conditional test on a specific feature (for example, new cases are classified as schizophrenia if their score on Linguistic irony is below 0.5 as indicated by the value in the branch between the two nodes). The main factors able to discriminate between the two classes (schizophrenia and healthy controls) in the generated tree are linguistic irony, Gricean maxims of linguistic communication, extralinguistic deceit, and extralinguistic sincere (direct and indirect) communicative acts. The strongest predictor for classifying patients with schizophrenia vs. healthy controls is *linguistic irony* (node 1): if the score on linguistic irony is below 0.5, a new case is classified as schizophrenia (with probability = 94.1%). The next decision point is *Gricean maxims of linguistic communication* (node 2) which, depending on whether its value is below 0.5 or above 0.5, leads to the evaluation of *extralinguistic deceit* (node 3) or *extralinguistic sincere (direct and indirect) communicative acts* (node 4), respectively. If *extralinguistic deceit* is below 0.8, the individual is classified as a patient with schizophrenia (probability = 92.1%), otherwise as a healthy control (probability = 75.1%). Similarly, if the score for *extralinguistic sincere (direct and indirect) communicative acts* is below 0.6, the individual is classified as a patient with schizophrenia (probability = 100%), otherwise as a healthy control (probability = 85.1%). Overall model Accuracy was 0.821 (SD = 0.118), Sensitivity was 0.758 (SD = 0.285), Precision was 0.910 (SD = 0.151), Specificity was 0.900 (SD = 0.175), and the area under the ROC curve (AUC) was 0.894 (SD = 0.143).

## Discussion

In the present paper, we provided a comprehensive multimodal assessment of communicative-pragmatic ability in patients with schizophrenia in order to investigate the communicative phenomena and expressive modalities most informative for discriminating between patients with schizophrenia and healthy controls. To this aim, we applied a DT analysis to identify the pragmatic features that best distinguish between the two groups.

First, we found that the overall performance of the DT model was good. Accuracy was 82%, indicating that the model was able to reliably distinguish between the two populations. Sensitivity was 76%, with 24 patients with schizophrenia classified correctly, and Precision was 91%, showing a low number (4) of false-positive errors.

The results showed that linguistic irony emerged as the most important pragmatic phenomenon for determining the class of a new case, i.e., the classification of an individual as a patient with schizophrenia or healthy control. Irony is one of the most investigated phenomena in the pragmatic literature, and a wide body of research has reported that irony comprehension is severely impaired in patients with schizophrenia^18,25,27,68,69^. A recent meta-analysis^63^ showed a large and robust difference in irony recognition between patients with schizophrenia and healthy controls (*d* = −1.22, *p* < 0.0001), thus suggesting a high level of impairment in irony comprehension in schizophrenia.

Understanding irony is a high-level linguistic task and requires the interplay of different cognitive functions. Previously, several studies found an association between irony comprehension and different cognitive functions, such as the theory of mind (ToM)^42^, i.e., the ability to infer the speaker’s mental states^70^, executive functions (EF)^71–73^, and level of intelligence^23,40,74^. More recently, some authors proposed that the additional cognitive effort required for irony comprehension, compared to other communicative expressions, may be due to the complexity of the inferential ability necessary to identify the ironic communicative intention (see refs. ^18,75–77^). Recent neuroimaging studies confirmed that comprehension of irony, with respect to literal statements, engages an extended bilateral brain network including several fronto-temporal and fronto-parietal areas, which underlies the cognitive effort related to the use of different cognitive functions such as ToM, executive controls, and inferential processes (e.g.,^78–81^). Irrespective of the specific cognitive substrates at the origin of patients’ deficits in irony recognition, previous studies clearly showed how irony comprehension is a high-level task that recruits an extended cerebral network corresponding to the interplay of different cognitive functions^78,81^. In line with this literature, the results of the present study confirm that, of the pragmatic features evaluated, the linguistic irony was the most complex for patients with schizophrenia to understand, and the most informative for distinguishing between patients and controls.

The second most important factor in classifying patients with schizophrenia or healthy controls in the DT model was the recognition of violation of the Gricean maxims of linguistic communication. Gricean maxims refer to the norms which regulate the discourse between two or more individuals, and serve as rules for rational and effective communication, by ensuring that the information provided by the interlocutors is as informative as necessary (maxim of quantity), the contribution is true (maxim of quality), relevant (maxim of relation), and clear (maxim of manner). More in detail, in the present investigation the items composing our experimental task assessed participants’ ability to recognize the interlocutor’s non-intentional violation of one of the Gricean maxims by providing a confused, or not precise, or not relevant or prolix contribution to the communicative interaction.

The difficulties encountered by patients with schizophrenia in appreciating Gricean maxims of communication are well known, with several studies reporting a pronounced impairment in the ability to attune to and recognize the maxims^10,20,23,35,36,69,82^, and a recent meta-analysis^63^ showing a large difference between patients with schizophrenia and healthy controls in tasks assessing the detection of violation of Gricean maxims (*d* = −1.33, *p* < 0.001). Frith and Corcoran^35^ evaluated the comprehension of Gricean maxims in individuals with schizophrenia, and found that patients exhibiting negative symptoms, such as anhedonia, reduced social drive, and loss of motivation, committed more errors than controls with all the maxims except the maxim of relation. Binz and Brune^82^ investigated the ability of patients with schizophrenia to adhere to the Gricean maxims of relation and quantity, and found significant differences between patients and healthy controls in these two maxims, with patients using more words than necessary and reporting irrelevant or unnecessary details. Mazza and colleagues^69^ found significant differences between patients with schizophrenia and healthy controls in recognizing non-intentional violations of all four maxims in conversational exchanges and reported that these differences correlated with ToM impairment. Further, some authors have proposed that non-intentional violations of the Gricean maxims of communication are associated with relevant clinical features of the disorder. For example, Abu-Akel^83^ claimed that positive symptoms, among which formal thought disorder, may be associated with incoherence, illogicality, and the tendency to provide irrelevant and unnecessary information, i.e., tangentiality. The results of the present study confirm that the difficulties in adhering to the social norms of communication, which result in frequent disruptions of communication in conversational exchange as reported by clinicians and relatives, are a key feature in schizophrenia. In addition, our results indicated that the first two tasks able to discriminate between patients with schizophrenia and controls, i.e., irony and violation of Gricean maxims, are both expressed through linguistic means. This datum is in line with a wide body of literature showing specific language impairment in patients with schizophrenia (e.g.,^11,25,40^). The remaining relevant tasks identified by the DT model in classifying patients with schizophrenia or healthy controls were the comprehension and production of sincere communicative acts (direct and indirect speech acts) and deceit expressed through the extralinguistic, i.e., gestural, modality. As regards the role played by sincere communicative acts in our results, the tasks used in the present investigation are composed of items investigating direct and indirect sincere communicative acts. Indirect acts are those by which the speaker communicates more than what s/he is actually literally saying to the listener^84^, as in the example “This soup is insipid” (example of unconventional indirect act) proffered in order to obtain the salt from the interlocutor. Several studies in the literature reported that patients with schizophrenia have difficulty with the comprehension of indirect speech acts^85,86^ and proposed that such difficulty is principally explained by a patient’s deficit in Theory of Mind^42^, i.e., the ability to conceptualize another person’s mental states^70^. A similar explanation holds for deceitful tasks, that are often used to investigate ToM difficulties in patients with schizophrenia^35,87–89^.

Moreover, empirical research in schizophrenia has traditionally focused on assessing language impairments, considered a hallmark of the disease ever since the first definitions of the disorder. However, it is only more recently that some studies have begun to report the presence of deficits affecting the extralinguistic, i.e., non-verbal modality. These studies found patients with schizophrenia to be impaired in non-verbal communication, especially in the production of communicative and social gestures^43,44,90^, and showed that these deficits cannot merely be accounted for by patients’ motor disorders^90^. Moreover, deficits have also been reported in the perception and recognition of communicative gestures, as well as in the recognition of facial expressions (for a review see refs. ^45,47,91^). Finally, deficits affecting non-verbal modalities have been found to be associated with functional outcome^92^. The present results are in line with theserecent evidence and point to the importance of focusing the assessment of pragmatic ability in schizophrenia not only on the linguistic modality, but also on the extralinguistic communicative modality.

Finally, we should also acknowledge the limitations of the present work. First of all, this is an exploratory analysis. ML methods benefit from larger sample size and different samples to perform out-of-sample validation, while the sample included in the present study is relatively small (*n* = 67). Further, schizophrenia is a heterogeneous disorder, and clinical samples can vary widely with respect to patients’ clinical features. Thus, the present results need to be replicated in future studies with larger samples and across different clinical profiles. Second, the pragmatic ability can be measured in different ways, and previous studies in the literature have used different batteries and tasks (e.g.,^17,39,40,86^). These tasks can vary widely with respect to the cognitive and inferential load. For this reason, the communicative features we found to be the most informative for classifying patients and controls need to be confirmed in future studies across different pragmatic tasks and contexts.

To conclude, pragmatic ability includes a wide range of different skills, all of which contributing to successful communication. While previous studies reported a wide array of communicative impairments in schizophrenia, it is hard to identify which of the skills affected are the most informative for distinguishing between schizophrenia and healthy controls. However, the identification of these features may be relevant as a valuable aid for clinicians throughout the diagnostic process, improving the effectiveness of rehabilitative treatment, and targeting future research. Further, recent studies reported that pragmatic and language impairments represent a risk factor for developing psychosis^5,93–95^, and thus the identification of the most distinctive pragmatic features is crucial for targeting early effective intervention to enhance these skills and which may diminish the risk. Indeed, some authors recently proposed pragmatic impairment as a marker for schizophrenia^21,61,96,97^. In this perspective, it will be fundamental to identify which pragmatic behaviors can most reliably be associated with schizophrenia. In this work, we moved a step in this direction, showing how, in our sample, some communicative features and expressive means, i.e., linguistic irony processing, adherence to and recognition of Gricean maxims of linguistic communication, and comprehension and production of extralinguistic sincere and deceitful communicative acts, were the most informative features for distinguishing between patients and controls. A deeper understanding of the relevance of these deficits in patients with schizophrenia will also be useful in order to promote the creation and implementation of rehabilitation programs specifically designed (see for example^65,66^) to help patients overcome such difficulties.

## Methods

### Participants

Thirty-two individuals with schizophrenia (seven females; age: *M* = 40.17; years; SD = 10.19; education: *M* = 10.59; SD = 2.45) and 35 healthy controls (six females; age: *M* = 39.46; SD = 10.95; education: *M* = 10.57; SD = 2.46) took part in the research. Patients and controls were matched for gender, education, and age (see Table 1). All patients with schizophrenia met the Diagnostic and Statistical Manual of Mental Disorders^98^ criteria for schizophrenia diagnosis. Inclusion criteria for patients were: (1) not in an acute stage: all patients were in the chronic stage of the illness and clinically stable (2) Italian native speaker (3) achievement of a cut-off score in the following neuropsychological tests in order to exclude the presence of severe cognitive or linguistic deficits: (a) Mini-Mental State Examination (MMSE^99^). Cut-off 24/30; (b) Token Test^100^. Cut-off 5/6; (c) Denomination scale of the Aachener Aphasie test (AAT^101^). Cut-off: no deficit. (4) must have provided their informed consent.

**Table 1:** Clinical details of patients with schizophrenia (*N* = 32).

	ID Patients with schizophrenia																															
	1	2	3	4	5	6	7	8	9	10	11	12	13	14	15	16	17	18	19	20	21	22	23	24	25	26	27	28	29	30	31	32
Sex	M	F	F	M	M	M	M	M	F	M	M	M	M	M	M	M	M	M	M	F	M	M	M	M	M	F	M	M	F	M	M	F
Age	56	48	44	23	35	53	30	40	38	53	30	31	24	43	26	35	45	22	27	49	50	47	43	57	47	24	47	25	56	30	47	46
Education (Yrs)	8	13	13	13	8	11	8	13	8	13	13	13	10	13	13	8	14	13	8	13	10	8	13	8	8	13	8	8	8	8	8	8
Treatment																																
Typical neuroleptics	•					•					•	•				•					•			•								
Atypical neuroleptics		•	•	•	•		•	•	•	•			•	•	•		•	•	•	•			•		•	•	•	•	•	•	•	
PANSS																																
Negative symptoms	10	21	17	13	35	17	42	18	7	19	33	16	15	42	20	16	21	15	11	18	24	29	8	11	32	25	22	14	11	42	29	12
Positive symptoms	12	9	9	13	22	18	42	29	10	25	34	23	12	31	18	14	10	10	17	18	26	20	25	18	29	23	11	16	7	42	13	9
General symptoms	30	51	32	39	62	24	94	41	32	36	88	48	37	69	31	32	40	29	40	35	58	38	8	32	64	53	48	24	16	94	40	26
Total score	52	81	58	65	119	59	178	88	49	80	155	87	64	142	69	62	71	54	68	71	108	87	33	61	125	101	81	54	34	178	82	47

Exclusion criteria were: (1) current and/or prior neurological disorder, (2) anamnesis of head injury, (3) history of substance abuse, (4) impaired hearing or vision. This study was approved by the ‘A.S.L. TO3-TO4-TO5 A.O.U. San Luigi Gonzaga ethics committee, protocol number: 0008187 and all the participants provided a written informed consent to take part in the study.

### Material and procedures

The participants were administered the Assessment Battery for Communication (ABaCo^102^), a validated tool showing good psychometric proprieties, i.e., content validity, high inter-rater agreement, and internal consistency^103,104^. The tool is theoretically grounded on the Cognitive Pragmatics theory^4^, it is available in Italian^105^ and has previously shown to be able to discriminate between patients with schizophrenia and controls^18,20,77^. The Battery includes five different scales assessing the comprehension and production of a wide range of pragmatic phenomena. The *linguistic scale* evaluates the comprehension and production of different communicative phenomena, i.e., basic speech acts, sincere communicative acts, deceit, irony, expressed using the linguistic modality. The *extralinguistic scale* assesses the same communicative acts, but expressed using the extralinguistic modality, i.e., gestures. The *paralinguistic scale* assesses the comprehension and production of those communicative aspects that complement the interaction, such as facial expressions, prosody, eye-gaze, etc. This scale evaluates communicative phenomena, i.e., basic communicative acts, communicative acts expressing an emotion, and paralinguistic contradiction. The *context scale* evaluates the adequacy of a communicative act with respect to the norms of discourse (i.e., Gricean maxims) and social norms of communication. The *conversational scale* assesses the ability to take part in a conversation appropriately, adhering to the topic and respecting turn-taking rules.

Taken together, the five scales of the ABaCo comprise 72 items in the form of live interviewer-interviewee interactions and 100 short clips (20–25 s each). These clips are shown to the participants using a laptop and their administration takes ~90 min. At the end of each clip, the examiner investigates the correct comprehension of the protagonist’s conclusive communicative act or else elicits the production of a communicative act in response to the protagonist’s utterance or gesture, and then asks specific open questions for each item. The specific pragmatic phenomena and skills assessed in each scale are described, with examples, in Table 2.

**Table 2:** Description of the structure of the Assessment Battery for Communication.

All items are scored as correct (1 point) or incorrect (0 points), based on precise coding rules that are set out in the ABaCo administration manual^105^. See also refs. ^77,102,106,107^, for a more detailed description of the administration and scoring procedures.

### Data analysis

We analyzed the data using a DT^108^ classifier in order to identify the pragmatic phenomena most relevant for discriminating between patients with schizophrenia and healthy controls. DT is a commonly used ML method for classifying and predicting a target variable based on multiple covariates. It is a classifier with a tree structure, where each node is either: (1) A *leaf node* which represents the final outcome of a series of decisions and indicates the value on the target attribute (class). For example, in our case, it indicates whether a new case is classified as a patient with schizophrenia or a control (target attribute: class). (2) A *decision node* that represents a conditional test on a specific feature. For example, in our case, if the feature considered is Linguistic irony, the decision node represents the threshold scores on Linguistic irony for which an individual is classified as a patient with schizophrenia or healthy control. For example, if the participant’s score on Linguistic irony (feature) is below or above 0.5, that individual is respectively classified as a patient with schizophrenia or healthy control. Each branch of the subtree represents a possible outcome of the test, with subtrees representing conjunctions of features (and the tests performed on these features, i.e., decision nodes) that lead, in the end, to the class attribute, i.e., whether a case is classified as a patient with schizophrenia or healthy control. For example, if we consider the decision node of Linguistic irony (see Fig. 1), this gives origin to two branches based on the test performed on this feature: first branch: if Linguistic irony is below 0.5 a new case is classified as a patient with schizophrenia; second branch: if Linguistic irony is above 0.5, a further test is performed on a different feature (Gricean maxims of linguistic communication), which in turn gives origin to two further branches, and so on until a leaf node is reached and a case is classified as a patient with schizophrenia or healthy control.

The *estimation criterion* in the DT algorithm is the selection of a feature to test at each decision node in the tree. The goal of the *estimation criterion* is to select the feature that is most useful for classifying the cases. A good quantitative measure of the worth of a feature is a statistical property called *information gain* that measures how well a given feature discriminates the cases based on the class to which they are attributed. This measure is used to select the best feature from among the possible candidates at each step of the growing tree.

In the present study we used the J48 algorithm (an implementation of Quinlan’s algorithm C4.5) in the Weka workbench for ML, version 3.8.3^109^ to generate the DT model. We estimated the generalization performance of the DT model using 10-fold cross-validation, which is a technique for evaluating predictive models by partitioning the original sample into a training set to create the model, and a test set to evaluate it. We reported the following performance metrics: Accuracy, Sensitivity, Precision, Specificity, and the area under the ROC curve (AUC). Accuracy refers to the proportion of the total number of classifications that were correct in both classes (SCZ and HC), Sensitivity gives the proportion of cases of schizophrenia classified correctly, Precision gives the proportion of cases classified as schizophrenia that was correct, and Specificity gives the proportion of control cases classified correctly. The ROC (Receiver Operating Characteristic) curve tells us how well the model can distinguish between the two classes (schizophrenia and healthy controls). ROC is a probability curve, and AUC represents a degree or measure of separability which tells how capable the model is of distinguishing between the two classes. Metrics presented are collected across all folds and are related to the test sets.

We performed additional analysis to assess whether pragmatic phenomena were able to predict symptoms and medications in the group of patients with schizophrenia only. These analyses are reported in Supplementary Information.

## Supplementary information

Role of medications and clinical features on pragmatic performance

## Data Availability

Please note that the datasets generated and/or analyzed during the current study are not publicly available for ethical reasons: Due to the anonymity guaranteed in the informed consent form at the time of data collection, data cannot be publicly shared, and are controlled by the A.S.L.To2 ethics committee. Qualified researchers who wish to request access to these data may contact *direzione.psicologia@unito.it* (Department of Psychology, University of Turin, Italy) or the corresponding author.

## Code availability

All experiments were performed on the Weka workbench for machine learning, version 3.8.3, available to download in https://www.cs.waikato.ac.nz/ml/weka/. The decision tree was the J48 classifier with the following parameters: confidence factor at 0.5 and the number of minimum instances per node (minNumObj) at 2. Details regarding the analysis are available upon request to rogeriosalvini@ufg.br
