# Supplementary material for: Multimodal assessment of communicative-pragmatic features in schizophrenia: a machine learning approach
Source: NPJ Schizophr. 2021 May 24;7:28. doi: 10.1038/s41537-021-00153-4 (PMC8144364; doi:10.1038/s41537-021-00153-4)
Supplement: Supplementary file 1 — Role of medications and clinical features on pragmatic performance [file 41537_2021_153_MOESM1_ESM.pdf]

Supplementary Information

Role of medications and clinical features on pragmatic performance

We performed a Machine Learning (ML) analysis to assess whether pragmatic phenomena were able to predict symptoms and medications in the group of patients with schizophrenia only. We split the group of patients with schizophrenia into two subgroups, i.e., high vs. low levels of symptoms, based on the median score on the Positive and Negative Syndrome Scale (PANSS) total, negative and positive scales. For what concerns medications, we classified the patients in two groups, i.e., those taking typical and atypical antipsychotic medication. We then applied Decision Tree (DT) analysis in order to identify the pragmatic phenomena most relevant for discriminating between patients with high vs. low levels of symptoms on the three PANSS scales (total, negative and positive), and between patients with schizophrenia taking typical and atypical antipsychotic medication. We found low accuracy for all the models (medication: 53.13%, PANSS total score: 34.4%, PANSS positive score: 46.8%, PANSS negative score: 56.3%), thus indicating a weak association between clinical symptoms and specific communicative phenomena.

Moreover, an additional analysis was run in order to assess the role that different medications may have on patients' performance in terms of the different communicative phenomena examined. We thus compared the performance of patients for the different communicative phenomena in the two subgroups, i.e., those taking typical and atypical antipsychotic medication, using an unpaired t-test. We did not find any significant difference between the two subgroups in any of the communicative phenomena investigated in the main analysis ($.156 < t < 1.241$, $.234 < p < .877$), thus suggesting that comprehension and production of the different communicative phenomena do not seem to be associated with any specific antipsychotic medication therapy.

We also performed a correlational analysis using Spearman's rank correlation coefficient to assess whether specific communicative phenomena may be associated with clinical symptoms (PANSS total, PANNS positive, PANSS negative). We found that total PANSS score significantly correlates with linguistic basic speech acts (BSAs) (Spearman's $r = -.531$, $p = .028$), Positive symptoms PANSS subscales score correlates with linguistic BSAs ($r = -.526$, $p = .030$) and linguistic irony ($r = -.476$, $p = .006$), and Negative symptoms PANSS subscales score correlates with extralinguistic BSAs ($r = -.497$, $p = .042$)

The correlations between the other communicative phenomena and clinical symptoms did not reach the significance threshold (alpha = .05).

These results suggest that the only communicative phenomenon reliably associated with clinical symptoms are basic speech acts. Basic speech acts are the easiest phenomena to be understood, as they represent very basic and prototypical types of speech acts (Kasher, 1991). Observing a negative association between these phenomena and symptoms is not surprising, as it indicates that patients with schizophrenia with more severe symptoms on the PANSS scale and subscales are also impaired in the most basic pragmatic tasks, unlike patients with low levels of symptoms who are only impaired in the more demanding pragmatic tasks.

Overall, the analysis reported above, showed that the associations between symptoms and pragmatic phenomena are weak, and the only phenomena more reliably associated with clinical symptoms (BSAs) are not among those selected by the decision tree classifier used in the main analysis to distinguish between patients with schizophrenia and healthy controls.
